# Rapid ELISA Using a Film-Stack Reaction Field with Micropillar Arrays

**DOI:** 10.3390/s17071608

**Published:** 2017-07-11

**Authors:** Yuma Suzuki, Kazuhiro Morioka, Soichiro Ohata, Tetsuhide Shimizu, Hizuru Nakajima, Katsumi Uchiyama, Ming Yang

**Affiliations:** 1 Graduate School of System Design, Tokyo Metropolitan University, 6-6 Asahigaoka, Hino, Tokyo 191-0065, Japan; yuma-suzuki@ed.tmu.ac.jp (Y.S.); ohata-soichiro@ed.tmu.ac.jp (S.O.); simizu-tetuhide@tmu.ac.jp (T.S.); 2 Department of Biomedical Analysis, School of Pharmacy, Tokyo University of Pharmacy and Life Sciences, 1432-1 Horinouchi, Hachioji, Tokyo 192-0392, Japan; kmorioka@toyaku.ac.jp; 3 Graduate School of Urban Environmental Sciences, Tokyo Metropolitan University, 1-1 Minami-Osawa, Hachioji, Tokyo 192-0397, Japan; nakajima-hizuru@tmu.ac.jp (H.N.); uchiyama-katsumi@tmu.ac.jp (K.U.)

**Keywords:** ELISA, microbioanalysis device, film-stack reaction field, circulating flow, theoretical equation

## Abstract

A film-stack reaction field with a micropillar array using a motor stirrer was developed for the high sensitivity and rapid enzyme-linked immunosorbent assay (ELISA) reaction. The effects of the incubation time of a protein (30 s, 5 min, and 10 min) on the fluorescence intensity in ELISAs were investigated using a reaction field with different micropillar array dimensions (5-µm, 10-µm and 50-µm gaps between the micropillars). The difference in fluorescence intensity between the well with the reaction field of 50-µm gap for the incubation time of 30 s and the well without the reaction field with for incubation time of 10 min was 6%. The trend of the fluorescence intensity in the gap between the micro pillars in the film-stack reaction field was different between the short incubation time and the long incubation time. The theoretical analysis of the physical parameters related with the biomolecule transport indicated that the reaction efficiency defined in this study was the dominant factor determining the fluorescence intensity for the short incubation time, whereas the volumetric rate of the circulating flow through the space between films and the specific surface area were the dominant factors for the long incubation time.

## 1. Introduction

Immunoassays, such as enzyme-linked immunosorbent assay (ELISA), are the gold-standard methods for the detection of biomolecules such as proteins and DNA fragments in immunological reactions with applications in numerous disciplines, including the diagnosis of infectious diseases [1,2,3]. ELISAs are usually performed in 96-well microtiter plates, which provide advantages such as high specificity, sensitivity, and the ability to carry out multiple assays in a parallel manner [1,2,3]. However, it takes a long time to complete the assays due to the large diffusion distance of the target biomolecules, the need for large volumes of reagents, and the lack of portability [4,5,6].

Currently, microbioanalysis devices (MBDs), such as micro-total analysis systems and lab-on-a-chip, are being developed to achieve the rapid detection of biomolecules thanks to the development of suitable microfabrication techniques [7,8,9,10]. Miniaturizing the biomolecule- incubation region in MBDs, called the reaction field, results in a short diffusion distance of biomolecules and a high specific surface area, which is the ratio of surface area to volume. Many researchers have developed microstructured reaction fields in MBDs to increase the surface area of a reaction field for high detection sensitivity and to limit the diffusion distance of biomolecules for a rapid reaction [11,12,13]. On the other hand, the amount of biomolecules adsorbed on the surface of a reaction field is reduced due to the low sample volume used in MBDs, resulting in low detection sensitivity [14,15]. Moreover, ELISAs are also performed by reaction fields such as microchannels in two-dimensional microfluidics chips [5,6]. However, the surface area of the reaction field is small and the increase of the surface area is limited by adding microstructures such as microbeads [12] and nanomaterials with high aspect ratio [13] to a reaction field. Hence, the two-dimensional reaction field is not enough to achieve high detection sensitivity with a rapid reaction and both a higher volumetric flow rate and a higher specific surface area with a small diffusion distance are necessary for the simultaneous pursuit of high detection sensitivity and rapid reaction in MBDs.

Our research group has developed a film-stack reaction field with a micropillar array for an ELISA device using 96-well microtiter plates. This reaction field is composed of several films stacked three-dimensionally in the reaction field and a magnetic sheet at the bottom of the reaction field. The surface area contributing to a reaction is increased by the stacked films, resulting in high detection sensitivity. Furthermore, there is an array of micropillars on the films, which additionally increases the surface area and limits the diffusion distance of biomolecules, contributing to high detection sensitivity and a rapid reaction. In the ELISA procedure, this reaction field in a well is rotated by a magnetic stirrer, which induces a circulating flow around the film-stack reaction field and through the inertial space between films in an ELISA procedure. Many biomolecules are rapidly transported to the reaction-field surface by the circulating flow. Moreover, a high volumetric flow rate through the inertial space in this reaction field can be effectively provided by the circulating flow with the limited solution volume. Therefore, the reaction time can be expected to be much reduced in addition to achieving higher detection sensitivity in ELISAs using this reaction field compared with the reaction field in the two-dimensional microchip. Additionally, the solution volume used in immunoassays is reduced by a few percent compared to that in traditional ELISA in general micro- fluidic devices such as microchannels for immunoassays [4,5,6]. However, the dead solution volume increases because of continuous solution supply to the microchannel for high detection sensitivity. On the other hand, ELISAs with film-stack reaction fields can achieve volumes less than half that used in traditional ELISAs and the dead solution volume decreases because of the circulating flow with the limited solution volume in wells. Therefore, the film-stack reaction fields can be expected to reduce more total solutions volume used in immunoassays compared with microfluidics devices including microchannels. Singh et al. [16] investigated the efficiency of this reaction field in ELISAs and demonstrated the increase of the detection sensitivity using the film-stack reaction field.

Furthermore, our previous study [17] showed the effect of the structural dimensions of a micropillar array in the film-stack reaction field on the fluorescence intensity of ELISAs by fluid-flow and particle-trajectory analyses by performing a computational simulation in addition to IgA ELISA tests for the optimized design of the film-stack reaction field. According to the computational simulation analysis in this previous study, the factors determining the fluorescence intensity in terms of the transport of biomolecules in the film-stack reaction field were identified, such as the surface area, the structural dimensions, the flow velocity, and the supplied number and diffusion distance of the biomolecules to the inertial space in the film-stack reaction field. However, the effect of the film-stack reaction field on the shorter incubation time for the rapid diagnosis was not investigated. The dependence of the incubation time on the fluorescence intensity of ELISAs might be related to the volumetric rate of the circulating flow in a well with the rotation of the reaction field. Additionally, the computational simulation analysis was not enough to quantitatively evaluate these dominant factors although this analysis presented these factors. Hence, their evaluation is necessary for the optimization of a film-stack reaction field and an ELISA procedure for the reduction of the incubation time. Nevertheless, the rotation of film-stack reaction fields by a magnetic stirrer is not stabilized because of the imbalance between the magnetic force, the gravity force of a film-stack reaction field, and the buoyancy force acting to the reaction field in a well. Furthermore, the rotation time of the reaction field has not been strictly controlled in the ELISA procedure. Therefore, the correct rotation control of the reaction field is required for the quantitative evaluation of the time dependence and the effect of the circulating flow.

In this study, a film-stack reaction field using a motor stirrer was developed to stabilize the rotation and for the easy control of the rotation speed and time of reaction. Furthermore, the dependence of the incubation time on the fluorescence intensity in the ELISA was investigated using this stirrer and the film-stack reaction field with micropillar arrays of different dimensions. Moreover, this study performed the theoretical modeling and analysis of the physical parameters related with the transport of biomolecules, such as the specific surface area, the diffusion time and distance, and the volumetric flow rate, in wells without and with film-stack reaction fields based on the computational analysis in our previous study [17] in light of the results of the ELISA tests for the optimization of the reaction-field design and the ELISA procedure with high sensitivity and a rapid reaction.

## 2. Materials and Methods

### 2.1. Fabrication of the Film-Stack Reaction Field with a Micro-Pillars Array

**Figure 1:**
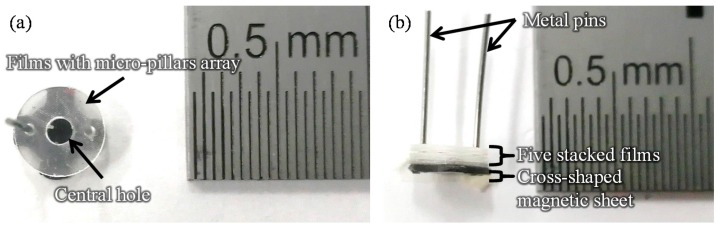
Images of the film-stack reaction field. (**a**) Top-view image. (**b**) Side-view image. The number of stacked film in a film-stack reaction field is five. Two metal pins fix five films and magnetic sheet. The outside diameter and central-hole diameter of the punched film are 5.0 and 2.0 mm, respectively.

**Figure 2:**
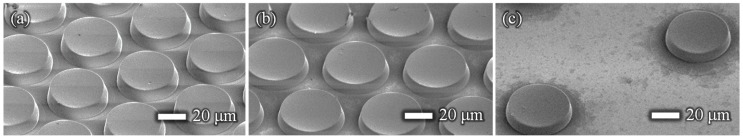
SEM images of the micropillar arrays on the base film. The diameter and height of the micro- pillars for all dimensions are 50 and 10 µm, respectively. The pillar gaps are (**a**) 5, (**b**) 10, and (**c**) 50 µm.

Figure 1 shows images of the film-stack reaction field. Film-stack reaction fields were fabricated by a nanoimprint process and a punch-press process for the molding of micropillars arrayed on base films, and the shaping and stacking of base films, respectively, in accordance with our previous studies [16,17]. A polyethylene terephthalate (PET) sheet and acrylic resin were used for the base film and micropillars, respectively. Micropillar arrays on PET films were fabricated using a nanoimprint technique. Five films with a micropillar array were automatically punched and stacked by a press machine. Five punched films were fixed by two metal pins. Moreover, a magnetic sheet, which was punched in a cross shape, was fixed at the bottom of the film-stack reaction field. The outside diameter and central-hole diameter of the punched film are 5.0 and 2.0 mm, respectively. Micropillar arrays with different dimensions were prepared to design a highly effective reaction field. Table 1 gives the structural dimensions of the micropillar array. Figure 2 shows scanning electron microscopy (SEM) images of micropillar arrays of different dimensions.

### 2.2. Experiment Setup

**Figure 3:**
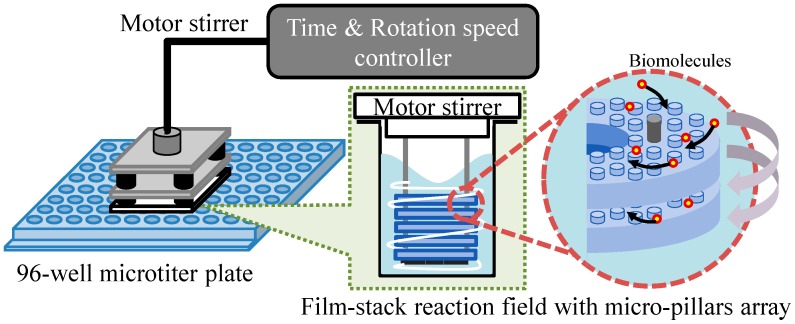
Schematic image of the experimental setup for the ELISA procedure.

**Figure 4:**
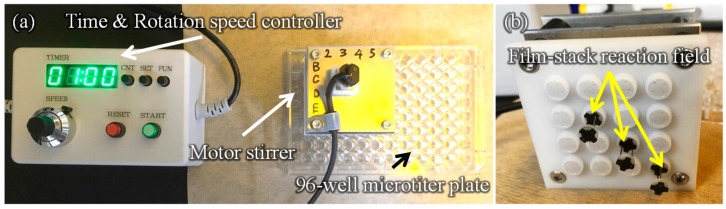
Images of the developed motor stirrer. (**a**) Whole image. (**b**) Bottom view of the rotated part in the motor stirrer. In the ELISA procedure, film-stack reaction fields are put on the bottom of the rotated part in the motor stirrer and this stirrer is set up on a 96-well microtiter plate to place reaction fields in wells.

Figure 3 presents a schematic image of the experimental setup for the ELISA procedure. This setup is composed of a 96-well microtiter plate, film-stack reaction fields and a motor stirrer as shown in Figure 4. This stirrer was developed to stabilize the rotation and for the easy control of the rotation speed and time of reaction. Furthermore, it can rotate sixteen reaction fields in parallel and perform multiple ELISAs for various biomolecules. In the ELISA procedure, film-stack reaction fields are put on the bottom of the rotated part in the motor stirrer and this stirrer is set up on a microtiter plate to place reaction fields in wells.

### 2.3. IgA ELISA

IgA ELISA was carried out using wells with or without film-stack reaction fields with micropillar arrays having different dimensions at room temperature. The procedure followed is the same as described in our previous study [17]. For the incubation of human IgA (Bethyl Laboratories, Inc., Montgomery, TX, USA) on the surfaces of wells and the film-stack reaction fields, 100 µL of 1% purified human IgA diluted in 0.1 mol/L carbonate buffer solution (CBS, pH = 9.5) was added to each well. In the wells with the film-stack reaction fields, these reaction fields were rotated by a motor stirrer for 1 min and left for 59 min. The well without the reaction field was left for 1 h. After the incubation, the human IgA solution was removed from each well and each well was washed with 300 µL of Tris-buffered saline (TBS, pH = 8.0) containing 0.05% Tween-20 (TBS-T). Then, blocking was performed using 200 µL of TBS containing bovine serum albumin (EMD Millipore Corp., Billerica, MA, USA, TBS-BSA) for 30 min. In the case of the wells with the film-stack reaction fields, these reaction fields were rotated by a motor stirrer for 1 min and left for 29 min. After the blocking, the TBS-BSA solution was removed from each well and each well was washed with 300 µL of TBS-T. Furthermore, for the incubation of horseradish peroxidase conjugated goat human IgA antibody (HRP-IgA, Bethyl Laboratories, Inc., Montgomery, TX, USA) in each well, 100 µL of 0.01% HRP-IgA diluted in TBS-BSA was added to each well. This step was performed with three incubation times of 30 s, 5 min, and 10 min to evaluate the effect of HRP-IgA incubation in each well. The reaction fields were continuously rotated during HRP-IgA incubation. After the incubation, the HRP-IgA solution was removed from each well and each well was washed with 300 µL of TBS-T. Finally, 100 µL of 1% Amplex^®^ Red (Thermo Fisher Scientific Inc., Waltham, MA, USA) solution diluted in 50 mmol/L phosphate-buffered saline (PBS, pH = 7.4) and hydrogen peroxide solution was added to each well. Then, the plates were developed in a dark place for 15 min, while the reaction fields were continuously rotated. After Amplex^®^ Red stop reagent (Thermo Fisher Scientific Inc., Waltham, MA, USA) was added to each well, 80 µL of the reaction solution was moved to another well in order to align the height of the solution level in all wells. The fluorescence intensity in each well was measured using a plate reader (SpectraFluor, TECAN, Männedorf, Switzerland). The rotating speed of the film-stack reaction field in each step was 1000 rpm, which is the maximum speed for the motor stirrer, which generates a sufficient circulating flow in a well.

### 2.4. Theoretical Modeling of Physical Parameters Related with Biomolecule Transport

#### 2.4.1. Geometric Model of Well and Space between Films

**Figure 5:**
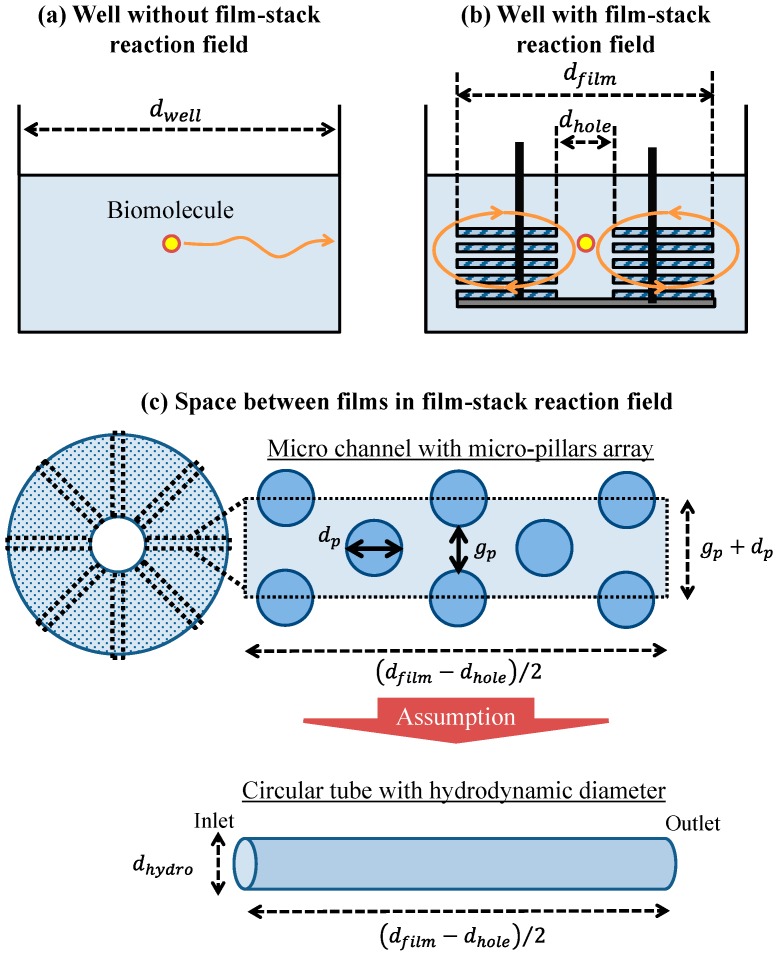
Geometric models of the well (**a**) with and (**b**) without the film-stack reaction field including the transport of one biomolecule. (**c**) Conceptual model of the fluid flow and biomolecule transport through the space between films. *d_well_*, *d_film_*, *and* *d_hole_* are the well diameter, the outside diameter of a film, and the central-hole diameter of a film, respectively. *g_p_* and *d_p_* are the gap between the micropillars and the pillar diameter, respectively. *d_hydro_* is the hydrodynamic diameter of a circular tube.

To verify the effect of the film-stack reaction field with a micropillar array on the fluorescence intensity in terms of physical phenomena, a theoretical analysis of the physical parameters related with biomolecule transport was performed. This analysis is based on the computational simulation of its transport in a film-stack reaction field as shown in our previous study [17]. Figure 5a,b shows the two-dimensional geometry models of the wells with and without the film-stack reaction field including the biomolecule transport in the theoretical analysis, respectively. The initial position of a biomolecule was assumed to be the well center. This biomolecule was assumed to move to the side wall of the well in a horizontal direction. In the well with the film-stack reaction field, the biomolecule was assumed to be transported through the space between films. Like in our previous study, the space between films was assumed to be composed of multiple microchannels with a micropillar array, as shown in Figure 5c. The number of microchannels per space between films *N_c_* is given by:

(1) $$N_{c}=\frac{\pi d_{hole}}{g_{p}+d_{p}},\;$$

where *d_hole_* is the central-hole diameter of a film, *g_p_* is the gap between micropillars, and *d_p_* is the pillar diameter. Furthermore, this channel was approximately modeled as a circular tube with a certain hydrodynamic diameter to simplify the theoretical analysis. The hydrodynamic diameter *d_hydro_* is expressed in terms of the dimensions of the micropillars as:

(2) $$d_{hydro}=\frac{2g_{p}h_{p}}{g_{p}+h_{p}}\;,$$

where, *h_p_* is the height of a micropillar. The initial position of a biomolecule was assumed to be the center of the circular-tube inlet. This biomolecule was assumed to move through the space between the films.

#### 2.4.2. Physical Parameters in Theoretical Analysis for the Well without the Film-Stack Reaction Field

The specific surface area of the well without the reaction field, *SS_w_*, is given by:

(3) $$SS_{w}=\frac{S_{w}}{V_{L}}\;.$$

In this equation, *S_w_* is the surface area of a well, and *V_L_* is the solution volume which is 100 µL for the ELISA procedure. Biomolecules in the well without a film-stack reaction field are transported by diffusion. The biomolecule arrival time at the side wall of the well, *t_w_*, is expressed by the diffusion time in Fick’s law as:

(4) $$t_{w}=\frac{{d_{well}}^{2}}{16D_{b}}\;,$$

where *D_b_* is the diffusion coefficient of a biomolecule given by $D_{b}=k_{B}T/6\pi \mu r_{b}$ (*k_B_* Boltzmann constant; *T* the atmosphere temperature; *µ* viscosity of the buffer solution in a well, *r_b_* is the biomolecule radius).

#### 2.4.3. Physical Parameters in Theoretical Analysis for the Well with the Film-Stack Reaction Field

The specific surface area of the well with the reaction field, *SS_f_*, is given by:

(5) $$SS_{f}=\frac{S_{w}+n_{f}S_{f}}{V_{L}}\;.$$

In this equation, *S_f_* is the surface area of a film and *n_f_* is the number of films per film-stack reaction field, which is five in the fabricated reaction field. Biomolecules in the well with a film-stack reaction field are transported by the convection flow generated by the rotation of the reaction field in addition to their diffusion. During the rotation of a film-stack reaction field in the well, the buffer solution flows through the space between the films in a laminar flow according to the analysis result of a computational simulation in our previous study [17]. Therefore, biomolecules can be assumed to be adsorbed on the film wall in the space between the films owing to their diffusion. The biomolecule-arrived time at the film wall in the space between films by its diffusion corresponds to the diffusion time of a biomolecule in the model of a circular tube with the hydrodynamic diameter *t_f,d_* as shown in Figure 5c, which is given by:

(6) $$t_{f,d}=\frac{{d_{hydro}}^{2}}{16D_{b}}\;.$$

Furthermore, the transit time of the biomolecules transport by the convection flow though the space between the films is provided by:

(7) $$t_{f,f}=\frac{d_{film}-d_{hole}}{2v_{f}}\;.$$

In this equation, *v_f_* is the average velocity of the convection flow in the circular tube with the hydrodynamic diameter. To investigate the dominant factor in the biomolecule transport in the circular tube such as the convection flow or its diffusion, Peclet number, *Pe*, which is the non-dimensional number in fluid mechanics expressing the ration of the diffusion time to the convection-flow time, was calculated as:

(8) $$Pe=\frac{t_{f,d}}{t_{f,f}}=\frac{v_{f}{d_{hydro}}^{2}}{8D_{b}\left(d_{film}-d_{hole}\right)}\;.$$

In the case of the convection flow for the biomolecule transport through the space between the films, this transport was evaluated on the basis of the volumetric flow rate in this space. To calculate the volumetric flow rate in the space between the films, the volumetric flow rate in the circular tube was calculated as:

(9) $$Q_{c}=v_{f}A_{c}=\frac{1}{4}\pi v_{f}{d_{hydro}}^{2}\;,$$

where *A_c_* is the diameter of the circular tube. Therefore, the volumetric flow rate in the space between the films was calculated by multiplying the volumetric flow rate in the circular tube and the number of micro channels per space between the films as:

(10) $$Q_{f}=N_{c}Q_{c}=\frac{v_{f}d_{hole}{\left(\pi d_{hydro}\right)}^{2}}{2\left(g_{p}+d_{p}\right)}\;.$$

Moreover, the volume of the buffer solution transiting through all the spaces between the films during the proteins incubation with the reaction-field rotation, *V_T_*, is calculated as:

(11) $$V_{T}=Q_{f}t_{i}\left(n_{f}-1\right)\;.$$

**Figure 6:**
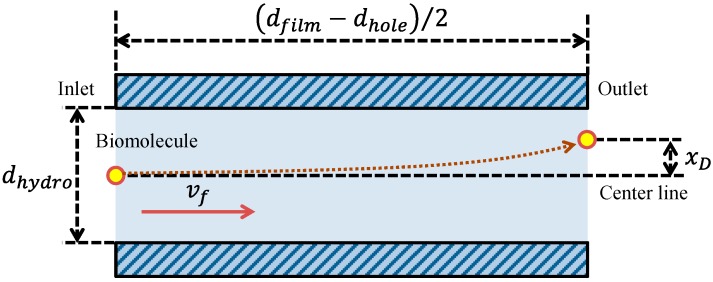
Model of the biomolecule trajectory in a circular tube with a hydrodynamic diameter as shown in Figure 5. *x_D_* is the diffusion distance of a biomolecule during its transport by the convection flow from the inlet to the outlet of the tube.

In this equation, *t_i_* is the protein incubation time. As another factor related to the fluorescence intensity in addition to the volumetric flow rate, the reaction efficiency of the film-stack reaction field was evaluated in this study. The reaction efficiency in this study corresponds to the probability of biomolecule adsorption on the wall in the space between the films with the circulating flow during the rotation of the reaction field. Here, to define this reaction efficiency, the simple model shown in Figure 6 was assumed. This model shows the biomolecule trajectory during its transport from the inlet to the outlet of a circular tube with a hydrodynamic diameter as shown in Figure 5. The diffusion distance of a biomolecule in the perpendicular direction to the tube axis during its transport by the convection flow from the inlet to the outlet of the tube, *x_D_*, is expressed by Fick’s law:

(12) $$x_{D}=\sqrt{4D_{b}t_{f,f}}=\sqrt{\frac{2D_{b}\left(d_{film}-d_{hole}\right)}{v_{f}}}\;.$$

Using Equation (12), the reaction efficiency in this model was defined as the ratio of the diffusion distance to half of the hydrodynamic diameter, which is the probability of the adsorption of the biomolecule on the tube wall during its transport by the convection flow from the inlet to the outlet of the tube. Therefore, the reaction efficiency, *E_R_*, is given by:

(13) $$E_{R}=\frac{2x_{D}}{d_{hydro}}=\frac{2}{d_{hydro}}\sqrt{\frac{2D_{b}\left(d_{film}-d_{hole}\right)}{v_{f}}}\;.$$

Moreover, the volumetric flow rate contributing to the reaction or the adsorption of the biomolecules in the wells with the film-stack reaction field is defined by multiplying the volumetric flow rate in Equation (8) and the reaction efficiency in Equation (13) as:

(14) $$Q_{f,E}=Q_{f}E_{R}=\frac{\pi^{2}d_{hole}d_{hydro}}{g_{p}+d_{p}}\sqrt{2v_{f}D_{b}\left(d_{film}-d_{hole}\right)}\;.$$

Table 2 shows the conditions for the calculation of physical parameters. The viscosity indicated in Table 2 is the water viscosity since the viscosity of the buffer solution was assumed to be similar to the water viscosity according to our previous study [17]. Furthermore, the average velocity of the convection flow for each hydrodynamic diameter with a rotation speed of 1000 rpm was calculated by the analysis of the computational simulation in our previous study.

## 3. Results

### 3.1. Fluorescence Intensity in IgA ELISA

**Figure 7:**
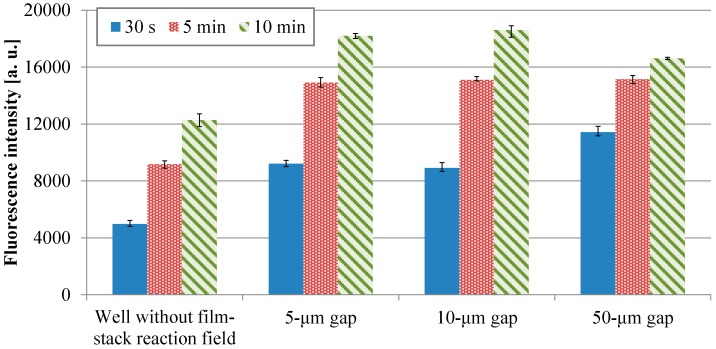
Fluorescence intensities of IgA ELISA in relation to HRP-IgA incubation time for wells without and with the film-stack reactions with the micro-pillars array having different dimensions. The trial number of experiments is three. Error bars are the maximum and minimum intensities.

Figure 7 shows the fluorescence intensities of IgA ELISA in relation to HRP-IgA incubation time for wells without and with the film-stack reactions with the micropillar arrays having different dimensions. For each incubation time, the fluorescent intensities in the wells with the film-stack reaction fields were higher than those in the wells without the reaction fields. The average intensities in the wells with the reaction fields for the incubation times of 30 s, 5 min, and 10 min were 2.0-fold, 1.6-fold, and 1.4-fold higher than those in the wells without the reaction fields, respectively. Thus, the film-stack reaction field with a motor stirrer was most effective with the short incubation time. Table 3 shows comparisons of the specific surface areas obtained using Equations (3) and (5), and the diffusion times obtained using Equations (4) and (6) between the wells without and with the reaction field. The specific surface area of the well with the reaction field was 3.1-fold higher than that of the well without it. The diffusion time of the well with the reaction field was five orders of magnitude less than that of the well without it. Furthermore, the difference in fluorescent intensities between the well with the reaction field of 50-µm gap for the incubation time of 30 s and the well without the reaction field for the incubation time of 10 min was 6%. Hence, these results indicated that the use of the film-stack reaction field with a motor stirrer can reduced the incubation time in ELISAs by 95% compared with the traditional ELISA using only 96-well microtiter plates.

**Figure 8:**
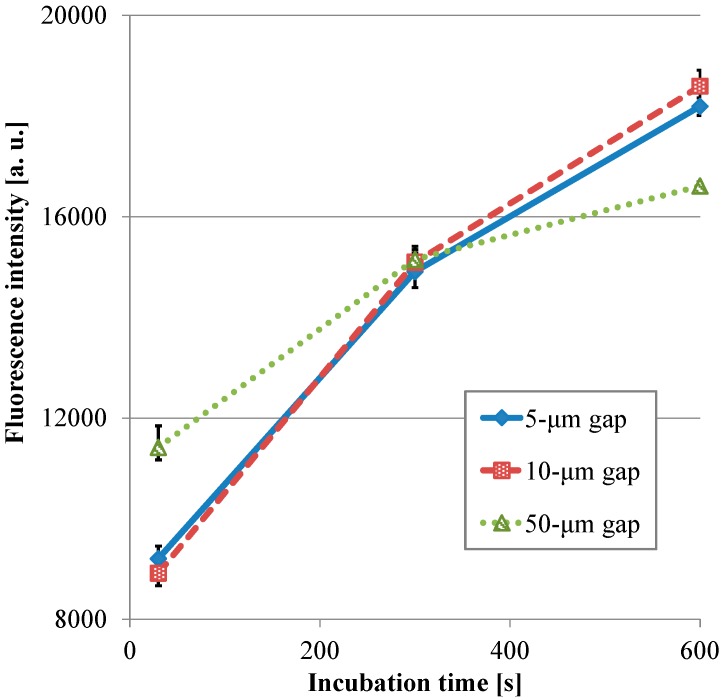
Transitions of the fluorescence intensity in the well with the film-stack reaction fields with the micro-pillars array having different dimensions in relation in relation to HRP-IgA incubation time. All fluorescence intensities are the same with Figure 7. Error bars are the maximum and minimum intensities.

Figure 8 presents the transitions of the fluorescence intensity in the wells with the micropillar arrays having different dimensions in relation in relation to HRP-IgA incubation time. For the long incubation time, the fluorescence intensity in the 10-µm gap was the highest and that in the 50-µm gap was the lowest among all the gaps. For the short incubation time, however, the fluorescence intensity in the 50-µm gap was the highest and that in the 10-µm gap was the lowest among all the gaps. Hence, the trend of the fluorescence intensity in the gap of the micropillar arrays in the film-stack reaction field was different for the long incubation time and the short incubation time. Furthermore, the fluorescence intensity in the 50-µm gap was highest and its gradient was smallest among all the gaps in the short incubation time. This means that the film-stack reaction with the 50-µm gap was closest to the optimum solution of the reaction-field design for rapid diagnosis with high detection sensitivity among all the gaps. To discuss this result, a theoretical analysis of the biomolecule transport with the fluid flow in a well was carried out, as described in the next section.

### 3.2. Theoretical Analysis of Physical Paramters Related with Biomolecule Transport

**Figure 9:**
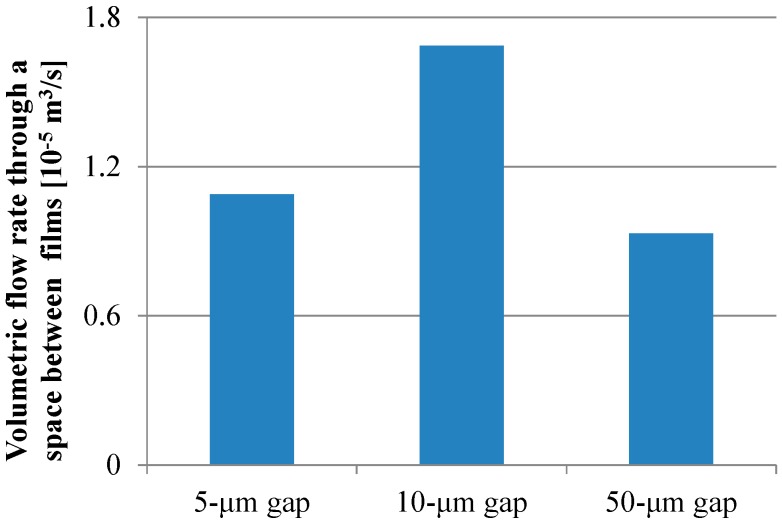
Volumetric flow rate through a space between films calculated using Equation (10).

For all dimensions of the micropillar arrays, Peclet numbers calculated using Equation (6) were more than 100, which indicated that the convection flow was the dominant factor in the case of the ELISA procedure using the film-stack reaction field with the rotation speed of 1000 rpm. Figure 9 shows the volumetric flow rate through a space between films calculated using Equation (10). The trend of the volumetric flow rate in the gap between micropillars was similar to that of the fluorescence intensity for the long incubation time, as shown in Figure 8. Moreover, the specific surface area of the reaction field with the 50-µm gap was lowest among all the gaps, as shown in Table 4. Therefore, the high volumetric flow rate and high specific surface area contributed to the high fluorescence intensity for the long incubation time.

**Figure 10:**
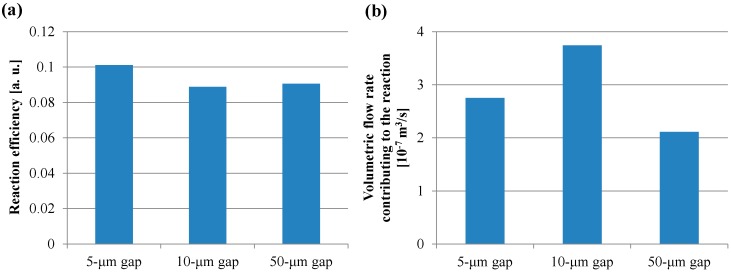
(**a**) Reaction efficiency of the film-stack reaction field calculated using Equation (13). (**b**) Volumetric flow rate contributing to the reaction or adsorption of the biomolecules in the wells with the film-stack reaction field calculated using Equation (14).

To verify the trend of the fluorescence intensity for the short incubation time shown in Figure 8, the volume of the buffer solution transiting through all the spaces between the films in the short incubation time was firstly calculated by Equation (11). The volume in the case of using the reaction field with the 50-µm gap for the incubation time of 30 s was the minimum and 1.1 L, which is more than ten-thousand-fold the buffer solution volume of 100 µL used in the ELISA procedure. This indicated that the sufficient circulating flow for proteins adsorption was generated by the rotation speed of 1000 rpm for the short incubation time. Figure 10a,b presents the reaction efficiencies of the film-stack reaction fields with the micropillar arrays having different dimensions obtained using Equation (13) and the volumetric flow rate contributing to the reaction or the adsorption of the biomolecules obtained using Equation (14), respectively. All the reaction efficiencies, however, were approximately 0.1 and the trend of the volumetric flow rate contributing to the reaction in the gap between films was unchanged from that of the flow rate shown in Figure 9 by the reaction efficiency. This might be due to the difference between the theoretical model in Figure 6 and the actual situation of the biomolecule adsorption on the wall in the space between the films. In the next section, this difference is discussed and the reaction efficiency given by Equation (13) is modified.

## 4. Discussion

**Figure 11:**
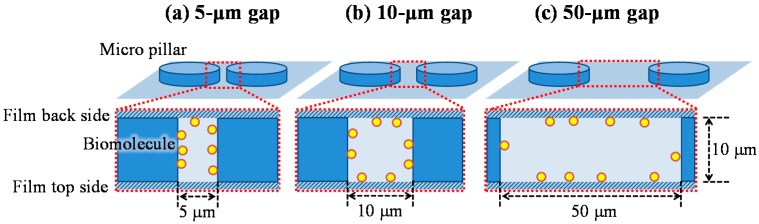
Schematic image of the situation indicating that the biomolecule adsorption on the wall in the space between films of the film-stack reaction field. (**a**) 5-µm gap. (**b**) 10-µm gap. (**c**) 50-µm gap.

Although the characteristic length of the reaction efficiency given by Equation (13) is the hydrodynamic diameter in the model of Figure 6, the biomolecules, in fact, preferentially adsorb on the wall closest to the biomolecules’ position. Figure 11 shows a schematic image of the situation indicating the biomolecule adsorption on the wall, which exists in the space between the films of the film-stack reaction field. While the biomolecules preferentially adsorb on the micropillar walls in the case of 5-µm gap, the biomolecules preferentially adsorb on the back and top sides of the film in the case of 50-µm gap. Hence, the reaction efficiency might need to be altered to the ratio of the diffusion distance to half of the minimum length in the structural dimensions of a micropillar array. The modified reaction efficiency, *E’_R_*, is given by:

(15) $${E^{\prime }}_{R}=\frac{2x_{D}}{L_{min}}=\frac{2}{L_{min}}\sqrt{\frac{2D_{b}\left(d_{film}-d_{hole}\right)}{v_{f}}},\;L_{min}=\{\begin{array}{cc} g_{p}\;, & g_{p}<h_{p}\\ h_{p}\;, & g_{p}>h_{p} \end{array}\;.$$

In this equation, *L_min_* is the minimum length in the structural dimensions of a micropillar array. When the gap between films is shorter than the pillar height, *L_min_*, is equal to the gap, while *L_min_* is equal to the pillar height when the gap is longer than the pillar height. Moreover, the volumetric flow rate contributing to the reaction obtained from Equation (15), *Q’_f,E_*, is expressed as:

(16) $${Q^{\prime }}_{f,E}=Q_{f}{E^{\prime }}_{R}=\frac{d_{hole}{\left(\pi d_{hydro}\right)}^{2}}{L_{min}\left(g_{p}+d_{p}\right)}\sqrt{2v_{f}D_{b}\left(d_{film}-d_{hole}\right)}\;.$$

**Figure 12:**
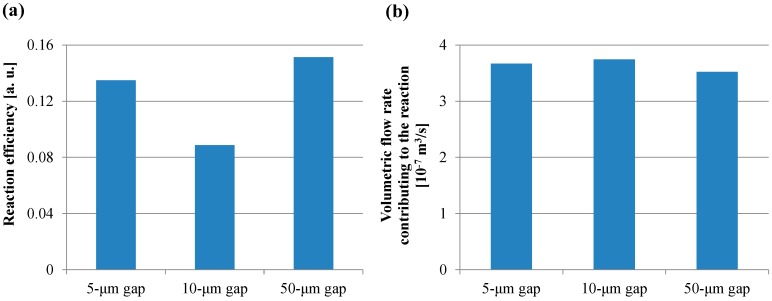
(**a**) Reaction efficiency calculated using Equation (15). (**b**) Volumetric flow rate calculated using Equation (16).

Figure 12a,b shows the reaction efficiency obtained using Equation (15) and the volumetric flow rate obtained using Equation (16), respectively. In Figure 12a, the trend of the reaction efficiency in the gap between the films was in agreement with that of the fluorescence intensity in the case of the short incubation time. On the other hand, all the volumetric flow rates shown in Figure 12b were comparable. Furthermore, the volume of the transited buffer solution which contributes to the reaction was calculated by assigning *Q’_f,E_* to the volumetric flow rate in Equation (11). The volumes of all the gaps between films in the case of the short incubation time were more than 40 mL, which is 400-fold higher than the buffer solution volume used in the ELISA procedure. Hence, the small diffusion distance and the low velocity with a sufficient circulating flow through the spaces between the films provided the high fluorescence intensity for the short incubation time in the ELISA procedure. Therefore, these results indicated that the reaction efficiency defined in this study was the dominant factor determining the fluorescence intensity for the short incubation time, whereas the volumetric flow rate and the specific surface area were the dominant factors for the long incubation time. Furthermore, the smaller diffusion distance provided by the structural dimension of a micropillar array and the lower rotation speed with the sufficient circulating flow through the spaces between the films can induce the higher fluorescence intensity for the shorter incubation time in addition to the higher specific surface area, which is achieved by increasing the number of films in the film-stack reaction field. Moreover, the equations proposed in this study can be used to quantitatively evaluate the function of the film-stack reaction field and are expected to be powerful tools for optimizing the design of the film-stack reaction field and the ELISA procedure to achieve high detection sensitivity and a rapid reaction.

## 5. Conclusions

A film-stack reaction field with a micropillar array using a motor stirrer was developed and IgA ELISA tests using this device were demonstrated. The effects of the incubation time of HRP-IgA on the fluorescence intensity in ELISA were investigated using film-stack reaction fields with micropillar arrays of different dimensions. The average fluorescence intensity of all wells with film-stack reaction fields was higher than that of wells without the reaction fields. In particular, the average intensity for the reaction field with the incubation time of 30 s was 2.0-fold higher than that of wells without the reaction fields. Furthermore, the difference in fluorescent intensity between the well with the reaction field of the 50-µm gap in the incubation time of 30 s and the well without the reaction field in the incubation time of 10 min was 6%. Thus, the incubation time in ELISA can be greatly reduced by the reaction field with a motor stirrer. On the other hand, the trend of the fluorescence intensity in the gap of the micro-pillars array in the film-stack reaction field was different between the short incubation time and the long incubation time. The theoretical analysis of the biomolecules transport with the fluid flow indicated that the reaction efficiency defined in this study was the dominant factor determining the fluorescence intensity for the short incubation time while the specific surface area was the dominant factor for the long incubation time. Furthermore, the smaller diffusion distance provided by the structural dimension of a micropillar array and the lower rotation speed with the sufficient circulating flow through the spaces between the films can induce the higher fluorescence intensity for the shorter incubation time in addition to the higher specific surface area, which is achieved by increasing the number of films in the film-stack reaction field. Moreover, the models and equations proposed in this study can be used to quantitatively evaluate the function of the film-stack reaction field and are expected to be powerful tools for optimizing the design of the film-stack reaction field and the ELISA procedure to achieve high detection sensitivity and a rapid reaction. The effectivity of the film-stack reaction field for immunoassays can be additionally presented to investigate solution volumes used in immunoassays, detection sensitivity, reproducibility, and applications on other immunoassays for biomolecules.

## Figures and Tables

**Table 1:** Structural dimensions of the micropillar arrays.

	Diameter [µm]	Height [µm]	Gap [µm]
(a)	50	10	5
(b)	50	10	10
(c)	50	10	50

**Table 2:** Conditions for the calculation of physical parameters in the theoretical analysis.

Characteristic Parameters for Physical Parameters	Values
Gap between micro pillars *g_p_* (µm)	5, 10, 50
Pillar diameter *d_p_* (µm)	50
Pillar height *h_p_* (µm)	10
Well diameter *d_well_* (mm)	7
Outside diameter of a film *d_film_* (µm)	5
Central-hole diameter of a film *d_hole_* (µm)	2
Surface area of a well *S_w_* (mm^2^)	93
Surface area of a film *S_f_* (mm^2^)	4.18 (5-µm gap), 4.00 (10-µm gap), 5.08 (50-µm gap)
Number of films per film-stack reaction field *n_f_*	5
Solution volume used in ELISA procedure *V_L_* (µL)	100
Biomolecule radius *r_b_* (nm)	6.5
Viscosity of buffer solution *µ* (Pa)	0.001
Atmosphere temperature *T* (K)	293.15
Average velocity of the convection flow in a circular tube with the rotation speed of 1000 rpm *v_f_* (m/s)	1.74 (5-µm gap), 1.01 (10-µm gap), 0.35 (50-µm gap)
Protein incubation time (s)	30, 300, 600

**Table 3:** Specific surface areas and diffusion times.

	Well without a Reaction Field	Average of Reaction Fields
Specific surface area (m^−1^)	930	2871
Diffusion time (s)	3.71 × 10^5^	1.07

**Table 4:** Specific surface areas calculated using Equation (5).

Structural Dimension of a Micropillar Array	Specific Surface Area *SS_f_* (m^−1^)
5-µm gap	3010
10-µm gap	2930
50-µm gap	2674
